# Sex Differences in Performance and Performance-Determining Factors in the Olympic Winter Endurance Sports

**DOI:** 10.1186/s40798-024-00792-8

**Published:** 2024-11-20

**Authors:** Guro Strøm Solli, Øyvind Sandbakk, Kerry McGawley

**Affiliations:** 1 Department of Sports Science and Physical Education, Nord University, Bodø, Norway; 2 School of Sport Science, UiT The Arctic University of Norway, Tromsø, Norway; 3 Department of Health Sciences, Swedish Winter Sports Research Centre, Mid Sweden University, Östersund, Sweden

**Keywords:** Gender, Cross-country skiing, Biathlon, Ski mountaineering, Speed skating, Nordic combined

## Abstract

**Background:**

Most sex comparisons in endurance sports have been derived from performance-matched groups of female and male athletes competing over similar distances within summer sports. Corresponding analyses of sex differences in winter endurance sports have not previously been conducted. In the Olympic Winter Games (OWG), the endurance sports include cross-country skiing (XCS), biathlon (BIA), Nordic combined (NC), ski mountaineering (SkiMo) and long-track speed skating (SpSk). The aim of this narrative review is to provide a comprehensive analysis of the sex differences in performance and performance-determining factors in the OWG endurance sports.

**Main Body:**

Sex differences in competition speeds are ~ 7–16% in XCS, 12–16% in BIA and 7–11% in SpSk, with race distances often shorter for women compared to men. No comparable data have been published for NC or SkiMo. Slower skiing speeds among women are associated with greater use of the diagonal and gear 2 sub-techniques in classic and skate skiing, respectively. In SpSk, slower skating speeds among women may be related to a less effective push-off being maintained throughout races. Laboratory data have revealed absolute and relative peak aerobic capacity to be 30–63% and 10–27% greater, respectively, in male versus female XCS, BIA, NC, SkiMo and SpSk athletes. There is limited evidence of sex differences in training characteristics, although women currently tend to complete more strength training than men in XCS and BIA. Of note, most data have been derived from studies performed in XCS, with almost no studies investigating sex differences in NC or SkiMo.

**Conclusions:**

This review provides a comprehensive overview of sex differences in performance and performance-determining factors within and between OWG endurance sports, which provides a scientific basis for designing training programs and future studies. Due to the lack of research investigating sex differences in NC and SkiMo, these sports, in particular, would be worthy of further attention.

**Key Points:**

- This narrative review provides a novel and comprehensive analysis of sex differences in performance and performance-determining factors in the Olympic winter endurance sports.
- Sex differences in competition speeds are ~ 7–16% in cross-country skiing, biathlon and speed skating, while no comparable data were available for Nordic combined or ski mountaineering.
- Since men have historically skied and skated over greater distances than women in cross-country skiing, biathlon and speed skating competitions, the “true” sex differences in performance are likely larger than the differences reported in the literature, therefore exceeding the differences typically reported for summer endurance sports.
- Most information about sex differences in Olympic winter endurance sports is based on studies performed in cross-country skiing. The conspicuous lack of information on Nordic combined and ski mountaineering warrants further research in these sports.

**Supplementary Information:**

The online version contains supplementary material available at 10.1186/s40798-024-00792-8.

## Background

Only 4% of the competitors at the first Olympic Winter Games (OWG) held in 1924 in Chamonix were women. At the Beijing 2022 OWG, female participation had risen to 45% and at the Milano Cortina 2026 OWG this proportion is expected to be 47%, with women competing for 43% of the 116 gold medals on offer [1]. Increases in female representation have been accompanied by a development in the professionalization of women’s sports and a reduction in sex differences in performance. In Olympic running events, for example, the sex gap in world record performances decreased from ~ 30% in the early 1920s to ~ 11% in the mid-1980s [2]. More recently, performance differences of 8–12% have been demonstrated between women and men in summer endurance sports [3, 4]. Physically, sex differences in endurance performance are primarily driven by high levels of testosterone produced by the testes during male puberty, which leads to differences in body size and body composition and higher hemoglobin concentrations in men versus women [3, 5, 6]. These factors are associated with differences in energy delivery capacity, with higher peak (VO_2peak_) and maximal (VO_2max_) oxygen uptake values and more skeletal muscle mass for generating metabolic energy in men [7–9]. However, sex differences in other performance-determining factors, such as markers of fractional utilization of VO_2max_ and work economy/efficiency, are less clear [6, 10]. In addition, our understanding of sex differences in biomechanical variables and training characteristics is currently limited.

Most sex comparisons in elite athlete populations have been conducted in performance-matched groups of women and men competing over equal distances within well-matured summer sports (i.e., sports with a relatively long history of both female and male participation) [6]. A corresponding analysis of sex differences does not currently exist for the OWG endurance sports (i.e., events that last longer than ~ 3 min and rely predominantly on aerobic energy delivery), which include cross-country skiing (XCS), biathlon (BIA), Nordic combined (NC), ski mountaineering (SkiMo) and long-track speed skating (SpSk). Many of the Olympic events within each of these sports are relatively new for women and in NC, for example, women are still not included at the Olympic level. Moreover, women compete over shorter distances than men in many of the events (Table 1). Due to the growth and development of women’s sport in recent decades, and a distinct need to increase knowledge about female athletic performance [11], an up-to-date analysis of sex differences in winter endurance performance and the associated mechanisms is justified. Accordingly, the aim of this narrative review is to provide a comprehensive analysis of sex differences in performance and performance-determining factors in the OWG endurance sports.

**Table 1:** Event details for each of the Olympic Winter Games Endurance Sports

Sport	Event	First included (year)		Distance^a^ (km)		Duration (min)		Additional information
		Women	Men	Women	Men	Women	Men	
XCS^b^	Sprint	2002	2002	1.0–1.8	1.0–1.8	2.5–3.5	2.5–3.5	Individual-start prologue followed by three knockout heats, each with six skiers
	Individual	1952	1924	10	10	22–25	20–23	Individual start
	Skiathlon	2010	2010	20	20	45–50	40–46	First half of the race in the classic technique, second half in the skating technique
	Mass start	1984	1924	50	50	130–145	115–130	–
	Team sprint	2006	2006	6 × (1.0–1.8)	6 × (1.0–1.8)	6 × (2.5–3.5)	6 × (2.5–3.5)	Two skiers alternating, each skiing three times
	Relay	1956	1936	4 × 7.5	4 × 7.5	4 × 19–22	4 × 17–20	Four skiers of the same sex skiing once each
BIA^b^	Sprint	1992	1980	7.5 (3 × 2.5)	10 (3 × 3.3)	20–23	22.5–26.5	Individual start, shooting prone then standing
	Pursuit	2002	2002	10 (5 × 2)	12.5 (5 × 2.5)	30–34	31–33.5	Start time based on sprint results, shooting two prone then two standing
	Individual	1992	1960	15 (5 × 3)	20 (5 × 4)	41.5–43	44.5–48	Individual start, shooting two prone then two standing
	Mass start	2006	2006	12.5 (5 × 2.5)	15 (5 × 3)	34–37.5	36–38.5	Mass start, shooting two prone then two standing
	Relay	1992	1968	4 × 6 (3 × 2)	4 × 7.5 (3 × 2.5)	4 × (17.5–19)	4 × (18.5–20.5)	Mass start, four same-sex biathletes each shooting prone then standing
	Mixed relay	2014	2014	2 × 6 (3 × 2)	2 × 7.5 (3 × 2.5)	2 × (16–18.5)	2 × (18–19.5)	Mass start, two women and two men, each shooting prone then standing
NC^c^	Individual large hill	*NI*	2002	–	10	–	23–25	Two jumps (hill size 110–184 m) followed by XC skiing
	Individual normal hill	*NI*	1924	–	7.5	–	17–19	One jump (hill size 85–109 m) followed by XC skiing
	Team relay	*NI*	1988	–	4 × 5	–	4 × 12–14	One jump for each athlete (hill size 110–184 m) followed by a skiing relay
SkiMo^d^	Sprint	2026	2026	–	–	3–4	3–4	Knockout heats, each with six skiers
	Mixed relay	2026	2026	–	–	–	–	Mass start, two women and two men
SpSk^b^	500 m	1960	1924	0.5	0.5	0.6–0.7	0.5–0.6	Starting in pairs (two runs, one race starting in the outer lane and one in the inner)
	1000 m	1960	1976	1	1	1.2–1.3	1.1–1.2	Starting in pairs (one run)
	1500 m	1960	1924	1.5	1.5	1.6–1.7	1.9–2.0	Starting in pairs (one run)
	3000/5000 m	1960	1924	3	5	3.9–4.2	6.0–6.3	Starting in pairs (one run)
	5000/10000 m	1988	1924	5	10	6.7–7.0	12.5–13.0	Starting in pairs (one run)
	Mass start	2018	2018	6.4	6.4	8.5–9.0	7.8–8.5	Mass start with 12–16 skaters starting at the same time with sprint points every 4th lap
	Team pursuit	2006	2006	2.4	3.2	3.6–3.9	2.9–3.2	Two teams of three same-sex skaters starting on each side of the track

*XCS* cross-country skiing; *BIA* biathlon; *NC* Nordic combined; *SkiMo* ski mountaineering; *SpSk* long-track speed skating; *NI*: not included

^a^Some of the event distances have been modified since their original inclusion in the Olympic Winter Games

^b^Based on distances and durations in World Cup races during the 2022/23 season

^c^Based on distances and durations at the 2022 Olympic Winter Games in Beijing

^d^Based on data from the World Championships in 2021

## Methods

**Fig. 1:**
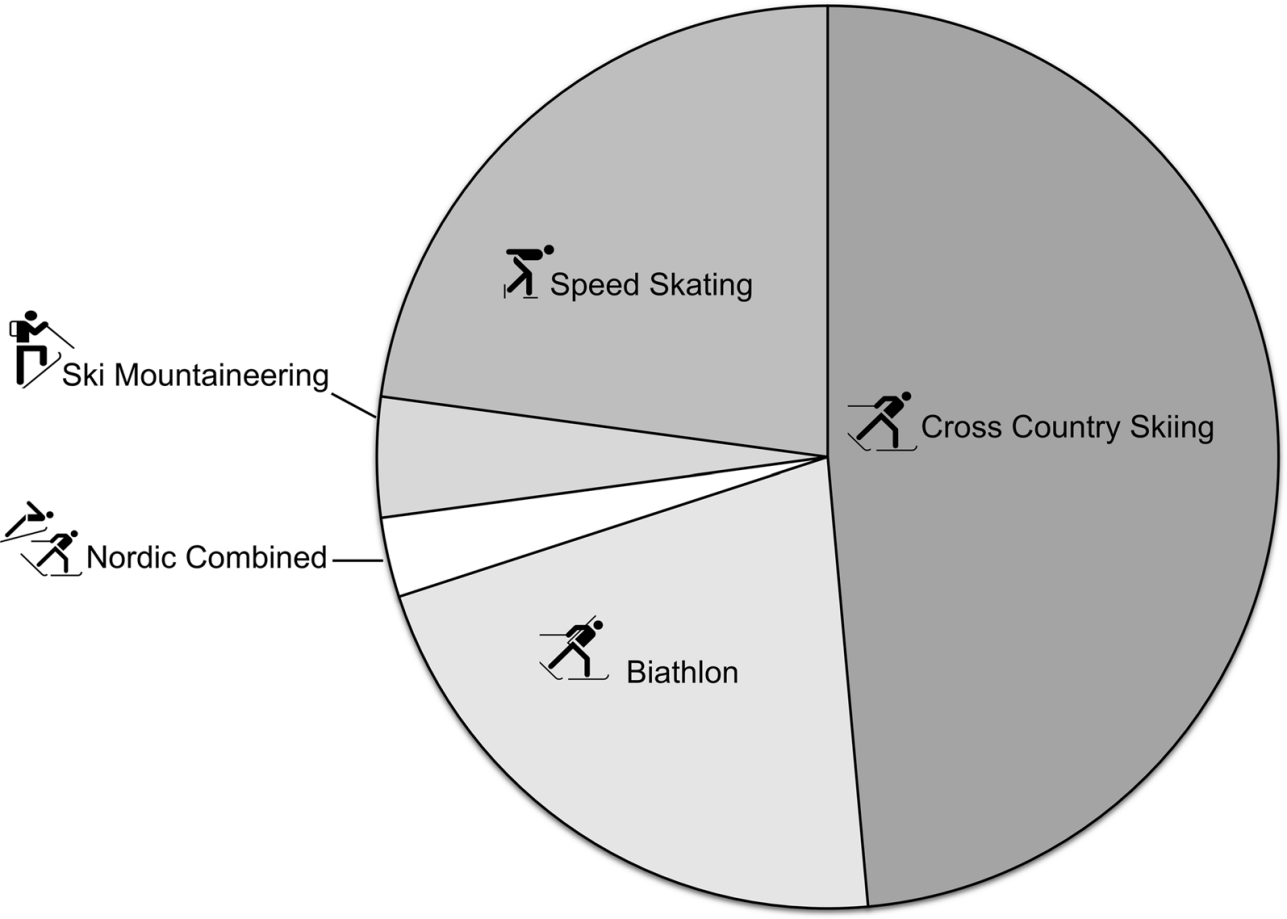
The distribution of winter Olympic endurance sports featured in the 62 studies included in this narrative review

Article databases including ScienceDirect, the US National Library of Medicine (PubMed) and SPORTDiscus were searched until 22nd December 2023 using the terms: gender OR sex differences AND cross-country skiing OR biathlon OR ski mountaineering OR speed skating OR Nordic combined. Additional relevant literature was then obtained from the reference lists of the published articles. Seven expert researchers with published studies involving at least one of the five included sports also suggested additional articles that could be considered for inclusion. The inclusion criteria were peer-reviewed scientific articles that contained: (1) information about sex differences in performance, performance-determining factors and/or training characteristics in one or more of the five included sports; (2) highly trained/national level (or higher) senior athletes (> 19 years old), which corresponds to tier 3 or higher using the participant classification framework of McKay et al. [12] and/or competitive junior athletes aged 15–19 years old. In total, 62 studies were included in this review and of these, 34 (55%) included information about athletes from XCS, 15 (24%) from BIA, 2 (3%; men only) from NC, 3 (5%) from SkiMo and 16 (26%) from SpSk (Fig. 1). Details of the screened papers are provided in Supplementary Table 1.

## Competition Formats of the Olympic Winter Endurance Sports

The five OWG endurance sports discussed in this review will account for 43 (37%) of the 116 gold medals (including 19 events for women, 22 events for men, and 2 mixed-sex events) at the Milano Cortina 2026 OWG. Nordic skiing as a form of locomotion features as a key component in XCS, BIA and NC and will play a role in 26 (60%) of the 43 OWG endurance events. The skating technique is used in XCS, BIA and NC events, while classic skiing is only performed in XCS. Differences in maturity of the various OWG endurance events, and fewer years of inclusion for women compared with men, may influence the current standards achieved by the two sexes. Although women are now included in four of the five OWG endurance sports (i.e., all except NC), women have typically competed in fewer events and over shorter distances and durations than men. The separate events within each sport for women and men are presented in Table 1, including information about the year they first featured in the OWG, the distances covered in each event and the typical event durations. In the following sub-sections, we explain the competitive formats and demands for female and male athletes in each of these sports.

### Cross-Country Skiing

The competition formats in XCS include sprint (involving a time-trial qualification followed by head-to-head knockout heats and a final), individual time trials, mass-start races, team sprints and relays (Table 1), all performed over undulating terrain, with ~ 50% of the race time spent skiing uphill. Until the 2022/23 season women competed over substantially shorter distances than men in the International Ski Federation (FIS) World Cup and as a result, distance-race durations were shorter and covered a narrower range for women (~ 25–75 min) compared to men (~ 35–120 min) [13]. FIS recently decided that women and men will compete over equal distances in the World Cup from the start of the 2022/23 season, making recent competition durations 10–15% longer for women compared to men. Therefore, Milano Cortina 2026 will be the first OWG where women and men will compete over equal distances in XCS.

### Biathlon

The competition formats in BIA include sprint and individual time trials, mass starts, team sprints and relays (Table 1). In BIA, Nordic skiing over undulating terrain in the skating style is combined with rifle shooting and throughout all competitions athletes must carry their rifle, weighing ≥ 3.5 kg, on their backs. All events are divided into three or five skiing loops interspersed with two or four shooting bouts, where the athletes stop to perform five shots in prone or standing positions. Women compete over distances that are ~ 25% shorter than men, resulting in slightly shorter competition durations in both the International Biathlon Union (IBU) World Cup, World Championships and OWG for women (~ 20–43 min) versus men (~ 23–48 min) [14].

### Nordic Combined

In NC, ski jumping and Nordic skiing using the skating technique are performed separately, but on the same day. Overall performance in NC is measured as a combination of the jump score and skiing time (FIS, 2022). In the individual (large and normal hill) and team relay events the athletes are seeded for the subsequent Nordic skiing discipline by the initial ski jumping component, which is scored by distance jumped and style. However, in the mass start event, which is included in the FIS World Cup program, the skiing component is performed before the jumping. Women are not currently included in the OWG (Table 1) but have competed in the World Cup since the 2020/21 season. Women typically compete in half the number of World Cup competitions as men, they only jump from the normal (and not the large) hill and the skiing distance is half that of the men’s (i.e., 5 vs. 10 km).

### Ski Mountaineering

In SkiMo, long uphill climbs are combined with descents on unprepared ski slopes and the competitions are either performed individually (sprint, vertical and individual) or in teams (team competition and relay) [15]. The sprint races consist of a qualification time trial followed by knockout heats (quarter-finals, semi-finals and a final) performed over a short course with a maximum of 80 m of ascent. Although women have traditionally competed over slightly shorter sprint courses, the durations required to win International Ski Mountaineering Federation (ISMF) World Cup sprint races over the past decade have been ~ 5–10% longer for women than men (~ 3.5 vs. 3.3 min) [16]. The vertical race is performed over similar courses for women and men, while the women’s individual race involves ~ 300 m less ascent/descent than the men’s equivalent. Of the five SkiMo competition formats, the International Olympic Committee (IOC) have included two events (sprint and mixed relay) for both women and men at the Milano Cortina 2026 OWG, where SkiMo is making its debut (Table 1).

### Long-Track Speed Skating

Individual SpSk is classified into sprint- (500 and 1000 m), middle- (1500 m) and long-distance (3000 and 5000 m for women, 5000 and 10,000 m for men) events (Table 1). There is also a mass start event where a maximum of 24 single-sex skaters start at the same time and compete over 16 laps (6400 m). Further, there is a team pursuit event, which involves teams of three women or three men completing 6 (for women) or 8 (for men) laps (2400 or 3200 m, respectively). In summary, speed skating competitions at the OWG range from ~ 36 s to 9 min for women (500–6400 m) and ~ 33 s to 12.5 min (500–10000 m) for men.

## Sex Differences During Competition

### Speed

Data from tier 4–5 XCS athletes competing in 10 km (women) and 15 km (men) individual national or international time-trial (i.e., individual start) races demonstrate that men ski 9% and 11–16% faster than women in the skating and classic styles, respectively [17–21]. When comparing 30 km (women) and 50 km (men) races in the skating style, the sex difference in speed was 7% for tier 3–5 athletes [22], while Andersson et al. [23] reported ~ 12.5% faster average speeds in a 1572-m simulated skating sprint competition for tier 3–4 men compared with women.

Based on 2011–2016 race data from IBU World Cup events in BIA, Luchsinger et al. [24, 25] observed 12 and 15% faster skiing speeds in sprint and individual races, respectively, for the top 10 men versus women. Similar data from IBU World Cup events between 2003 and 2019 revealed ~ 15 and 13% faster skiing speeds during the first lap for the top 20 men compared to women in sprint and individual races, respectively [26]. Furthermore, Björklund et al. [27] reported 14 and 15% faster skiing speeds during IBU World Cup pursuit and mass start races, respectively, for the top 3 women versus men analyzed between 2003 and 2019.

No studies have investigated sex differences in skiing speeds in NC. For this review, we analyzed the isolated skiing times for NC in the 2021 and 2023 World Championships and found that the top 3 men skied ~ 14–18% faster over 10 km than the top 3 women skied over 5 km. In SkiMo, there was a 21–25% speed difference in the first uphill skiing section and a 25–33% speed difference in the second uphill running section between the female and male winners of two ISMF sprint World Cup competitions [28]. However, differences in the competition tracks for women versus men made comparisons of overall competition speed impossible.

In SpSk, ~ 10% faster average speeds were reported for men compared to women over the 1500-m and 5000-m races during the Calgary 1988 OWG [29, 30]. Data from the International Skating Union (ISU) World Cup competitions during the 2007–2009 seasons revealed similar results, with 10.5, 9.8 and 9.1% faster speeds in the top 10 men compared to women in the 1000, 1500 and 5000-m races, respectively [31–33]. Contrary to this pattern of reduced sex differences as race distance increases, an analysis of SpSk world record performances has shown an increase in sex differences with race distance (i.e., 7 and 11% differences for 500-m and 5000-m races, respectively) [3]. No published analyses of sex differences in the relatively new SpSk mass start competition format are currently available.

In summary, the reported sex differences in average skiing/skating speeds range between ~ 7–16% in XCS, BIA and SpSk. We calculated 14–18% differences in NC skiing speeds, while no comparable differences for sex differences in overall competition speed could be calculated for SkiMo. Therefore, future studies could focus on analyzing sex differences in skiing speeds in NC and SkiMo. Notably, men have skied 10–70% further than women in XCS, BIA and NC competitions studied to date, indicating that the “true” sex differences in these sports would likely be larger than currently reported. Accordingly, sex differences in many of the OWG endurance sports likely exceed the 8–12% differences reported for summer endurance sports [2, 3, 34]. The exact extent to which differences in race distance, duration and ascent influence sex differences in performance remains to be examined under controlled experimental conditions.

### Pacing Strategies

In XCS, a general pattern of reduced lap-to-lap speeds (i.e., a positive pacing strategy) has been reported for tier 3–5 women and men in individual-start competitions [18, 21]. Losnegard et al. [35] reported that the fastest male skiers were able to maintain their lap speed better than slower male skiers (over 15 km), while no corresponding difference was found between faster versus slower women (over 10 km). Pacing strategies in a 21.8-km mass-start XCS competition have been examined in national-level men [36], but no analyses of mass-start competitions have included women. Likewise, no analyses of sex differences in sprint XCS performance (i.e., from the time-trial qualification and through the head-to-head knockout heats and final) have been published. These are areas worthy of future research, as sex comparisons would shed light on the differences that may exist between women and men in race tactics over different race formats and distances, which could support the development of sex-specific training and preparation strategies.

Biathletes of both sexes tend to adopt a J-shaped pacing strategy during sprint and individual IBU World Cup competitions, with lap-to-lap speeds decreasing after the first lap and fastest in the final lap (i.e., after the last shooting bout) [24, 25]. Pursuit races elicit a reverse J-shaped pacing pattern, with the first lap being fastest and the fourth lap slowest, followed by an increase in speed on the final fifth lap [27]. In contrast, mass-start races begin with a slow first lap and the second lap is fastest, although the fastest women and men are able to increase their speed on the final fifth lap [27]. Overall, similar lap-to-lap (i.e., “macro-pacing”) patterns are observed for both sexes, independent of the differences between competition formats and associated event demands.

Due to the varying terrain in XCS, BIA and NC, the pacing strategies employed by athletes over specific course sections (e.g., uphill, flat and downhill sections), which has been defined in the scientific literature as “micro-pacing”, is important [37]. In distance races (10 km for women and 15 km for men) in skating and classic XCS [17], and a simulated sprint race using the skating technique [23], sex differences in speed were larger in uphill compared to flat terrain (11–19 vs. 6–12%). However, two other studies investigating classic distance XCS races reported slightly larger sex differences on intermediate inclines compared to steeper uphill terrain (20–21 vs. 17–19%) [18, 20]. In a distance competition in skate XCS with tier 3 athletes, Staunton et al. [38] showed that the course sections where the fastest athlete gained most time over the slowest athlete differed for women (i.e., a flat section) and men (i.e., an uphill section), which might reflect different micro-pacing strategies adopted by the two sexes. Terrain-induced sex differences in performance are likely due to a combination of increased upper-body power in men compared to women and greater lean mass and/or reduced fat mass in men, which affects power-to-weight ratios between the sexes [39] (these explanations are discussed in more detail in "Sub-technique selection in Nordic skiing" and "Anthropometry" sections).

Two recent SkiMo studies have reported that the fastest athletes increased their uphill speeds more from the qualification to the finals compared with the slower athletes [16, 28]. However, sex differences in pacing patterns have not yet been examined in NC or SkiMo, and the general lack of performance analyses in these two sports highlights the need for more research. By contrast, pacing patterns in SpSk have been of scientific interest for decades, using both simulation [40] and experimental studies (for a review, see Konings et al. [41]). For tier 3–5 women and men the general pacing pattern observed in SpSk events ranging from 1500 to 10000 m [31, 32, 42] is characterized by an initial acceleration to maximum speed in the first part of the race followed by a progressive decay in lap times (i.e., a positive pacing strategy). These studies showed that longer distance races have slower relative opening speeds and more even pacing than shorter distance races and that superior athletes of both sexes were able to maintain speed better than their slower counterparts. This pattern is similar to running, where more even pacing strategies are adopted during longer distances (i.e., 800 vs. 5000 m), and superior athletes are better able to maintain their pace [43, 44]. The relatively new SpSk mass start competition format is more tactical and the athletes often start at a slow pace, then speed up for the intermediate sprints and finish with two very fast laps [45].

In summary, macro-pacing strategies appear to be relatively similar for women and men, mediated by the demands of the event rather than the sex of the athlete. Regarding micro-pacing strategies in XCS and BIA, terrain-specific sex differences are likely associated with differences in physical characteristics (i.e., upper-body power and body composition) between women and men. Future research could examine sex differences in pacing strategies in mass-start and sprint XCS events, mass-start SpSk events, all NC and SkiMo events, and female and male race tactics across all OWG endurance sports.

### Sub-technique Selection in Nordic Skiing

Four major sub-techniques characterize the classic and skate Nordic skiing styles, respectively [46]. Sub-techniques can be compared with bicycle or car gears, in which the movement pattern is adapted to account for changes in speed and terrain. In the classic style, skiers use the herringbone technique, diagonal stride (DIA), double poling with kick (DK) and double poling (DP). In the skating style, skiers use gear (G) 2–5 (also referred to as V1, V2, V2a and skating without poles). Turning techniques and the downhill tuck sub-technique are also used in both styles. The use of different sub-techniques is influenced by a combination of speed, incline, physical strengths, and individual technical preferences [46].

During a classic XCS international time-trial race (10 and 15 km for women and men, respectively), Solli et al. [18] reported that tier 4–5 women spent 15% more of their race time using DIA and 15% less time using DP compared to men. Similar sex differences were reported by Stöggl et al. [20] for tier 3–5 athletes in an intermediate terrain section (22 m at 3.5°) in the same competition format. When using the skating style, Andersson et al. [23] reported that the 20% sex differences in uphill skiing speeds were associated with women using a lower gear (G2 vs. G3) more frequently than men during a simulated sprint competition. Consistent with this, Ardigó et al. [22] reported greater use of G2 in women compared to men during a transition section (21 m with a 5–8° incline) in a long-distance XCS race (30 and 50 km for tier 3–5 women and men, respectively). Similar findings have been reported in BIA, with women spending more time and covering more distance using G2 versus G3 compared to men, both when skiing with and without a rifle [47].

The above-mentioned sex differences in sub-technique selection are mainly explained by the higher speeds achieved by men, which allows them to ski a higher proportion of the competition tracks using higher gears [18, 20, 22, 23, 47]. Less frequent use of DP and G3 by women may also result from the greater upper-body contribution in these sub techniques, since sex differences in upper-body power are greater than for the lower body [48]. The enhanced ability of men to utilize DP in all terrain was demonstrated by Stöggl et al. [49], who reported increased sex differences in speed during a simulated 5.3-km on-snow classic XCS race when tier 3–5 female and male skiers were limited to using DP exclusively, compared to freely selecting their sub-technique. In addition, a higher number of transitions between sub-techniques is observed in female compared to male skiers over the same competition track terrain sections [18, 20]. A proposed reason for this is that women typically ski at speeds closer to their thresholds for sub-technique transitions, leading to more frequent transitions compared to men [20]. This point needs further investigation but could indicate that it is more important for female than male athletes to master transitions between sub-techniques.

Overall, women tend to use DIA and G2 more frequently, and DP and G3 less frequently, in classic and skate skiing, respectively. This is due to the higher skiing speeds achieved by men and their greater upper-body power. Whether female skiers would benefit from replicating men when it comes to sub-technique selection, or whether they should use other strategies to improve their performance, remains to be elucidated. Future research investigating the transitions between sub-techniques in women and men would help gain further insight into this aspect of Nordic skiing. Moreover, a large imbalance in the research literature was identified, with almost all data derived from XCS, only one study from BIA and no studies in NC.

### Biomechanical Characteristics

The speeds of locomotion in the OWG endurance sports can be expressed as the product of cycle length (CL) and cycle rate (CR). In XCS distance races, higher speeds achieved by male versus female tier 3–5 athletes are related to a 15–26% longer CL in men, while CR is similar or slightly higher (by up to 9%) in women [18–20, 22, 23]. These findings are supported by Sandbakk et al. [39, 48], who reported a 21–25% longer CL but no differences in CR in male versus female tier 4–5 XCS sprinters during maximal treadmill roller skiing using DP and G3. While these sex differences in CL are connected to the higher skiing speeds in men, Jonsson et al. [19] also observed sex differences in pole and joint angle kinematics when accounting for differences in DP speed. For example, these observed differences included a smaller hip angle at pole plant and pole-off, a smaller distance between the pole tip and the toecap at pole-off, and a shorter poling distance (i.e., distance covered when the poles were in contact with the snow) for each cycle in female compared to male tier 4–5 skiers during a 22-m flat section in an individual XCS distance race.

In BIA, Jonsson Kårström et al. [50] observed minor sex differences in the range of motion (i.e., the difference between maximal and minimal angles) in joints, skis and poles during treadmill roller skiing at simulated race speeds. However, no data on sex differences in CL or CR are available for BIA. An interesting focus of future research could be to determine whether the greater relative mass of the rifle for women relates to sex differences in CL, CR and kinematic characteristics in BIA compared to skiing without the rifle.

In SpSk, the major performance-determining kinematic characteristics are reported to be the trunk angle (i.e., the position of the trunk in relation to the horizontal plane), the pre-extension knee angle (i.e., the knee angle before the start of the push off) and an effective directed push-off, reflected by a small angle between the push-off leg and the horizontal plane [51–54]. Studies seem to be consistent in reporting no sex differences in trunk angle [30, 54, 55], while a smaller pre-extension knee angle has been reported in tier 3–5 male compared to female athletes [30, 55]. In contrast, Noordhof et al. [54] observed no sex differences in pre-extension knee angle in tier 4–5 athletes. The difference between the earlier and more recent findings is likely due to greater relative improvements in female SpSk between the 1980s and early 2000s. An interesting observation from Noordhof et al. [54] was that male speed skaters were able to maintain a more effective push-off (i.e., a smaller push-off angle and a smaller increase in the push-off angle) compared with women throughout 1500 and 5000-m races. This might be an area worthy of further attention for female athletes and their coaches, to improve technical execution and SpSk performance.

Overall, there are limited data on biomechanical differences between sexes in XCS, BIA and SpSk and no studies have investigated sex differences in NC or SkiMo.

### Shooting Performance in Biathlon

Three of the sports included in this review involve non-endurance components (i.e., rifle shooting in BIA, ski jumping in NC and downhill skiing in SkiMo). While no information about sex differences in ski jumping and downhill skiing in NC and SkiMo, respectively, are available, some studies have investigated sex differences in shooting performance in BIA. Shooting performance is a crucial component of overall BIA performance and includes the time spent shooting and the number of hit/missed targets. During the IBU World Cup sprint and individual competitions between 2011 and 2016 the top 10 women spent ∼ 6% more time shooting than the top 10 men [24]. Moreover, Björklund et al. [26, 27] reported 7–10% shorter range times (i.e., the time between entering and exiting the shooting range) for men versus women during sprint, individual, pursuit and mass start races between 2012 and 2019. Whether sex differences in skiing speed (in and out of the range), the relative weight of the rifle or other physiological or psychological factors influence the observed sex differences in shooting and/or range times remains uncertain. These possibilities reflect potential areas for future research that may help support development among female biathletes, in particular.

Sex differences have been observed in BIA shooting accuracy, measured as hit/missed targets during prone and standing shooting [25, 56]. However, the differences are inconsistent between studies and across seasons. Relatively small sex differences (0.8 ± 2.2%) have been observed in Olympic and Paralympic rifle shooting performance, with better visuospatial abilities in males suggested as a potential explanation [57]. However, the extent to which the additional skiing component in BIA influences sex differences in shooting accuracy requires further investigation.

## Sex Differences in Performance-Determining Variables

### Performance in Laboratory Tests

A recent laboratory-based study of XCS athletes revealed 23, 24 and 17% longer distances covered by performance-matched adolescent (~ 15 years), junior (~ 18 years) and senior (~ 28 years, tier 4) male versus female athletes, respectively, during a 3-min uphill treadmill test using the G2 sub-technique on roller skis [58]. Similarly, junior male XCS athletes completed a 600-m (~ 3-min) laboratory-based treadmill time-trial test in 21% less time than their female counterparts when roller skiing using the DIA sub-technique [59]. These 17–24% sex differences in laboratory tests are somewhat higher than the differences in average competition speeds for women versus men [17–23]. The reason for this is likely due to the short test durations (~ 3 min) and the use of relatively steep uphill gradients in the test protocols, which typically favor men (see "Pacing strategies" section). As has been observed on snow, sub-technique use also influences the magnitude of the sex differences in laboratory tests. For example, Sandbakk et al. [48] reported progressively larger sex differences in peak speed obtained during an incremental treadmill test as the contribution from poling (i.e., the upper body) increased, with 14, 17 and 20% sex differences observed for DIA, G3 and DP, respectively.

In SpSk, sex differences of 46–60% have been reported for absolute mean power output (W) during a 150-s maximal cycle ergometer test in junior speed skaters [60–62]. These sex differences were reduced to 13–23% when scaled to body mass (W·kg^−1^) and to just 1–2% when scaled to lean body mass (W·kg LBM^−1^) [60]. Comparable sex differences in mean power output in W (42–48%) and W·kg^−1^ (8–15%) were observed in a 30-s maximal cycle ergometer (Wingate) test [60, 62], while two other studies reported somewhat higher sex differences (20–30%) in mean power output relative to body mass [63, 64]. The reason for the inconsistent findings between studies is uncertain, but is likely related to methodological differences (e.g., the test protocols and equipment used), the inclusion of sprint versus all-round speed skaters and/or differences in how closely performance was matched between the sexes within studies.

Overall, the sex differences in laboratory-based roller skiing tests in XCS are ~ 14–24%, with larger sex differences observed in short duration uphill tests and tests where the upper-body contribution is larger. In SpSk, most studies report sex differences in mean power output of 43–60% (in W) and 13–15% (in W·kg^−1^) during 30- and 150-s maximal cycle ergometer tests, which are primarily explained by the higher lean body mass (LBM) in male athletes. However, differences in test protocols, equipment, and participant characteristics make valid comparisons between studies difficult. To date, no studies have compared sex differences in laboratory-based performance in BIA, NC or SkiMo.

### Anthropometry

Carlsson et al. [65] reported a 41% higher absolute LBM in tier 3 male compared to female XCS athletes. This study also showed large to very large positive correlations between absolute LBM (for total, upper- and lower-body mass) and XCS sprint prologue performance in women and men. However, for distance performance this positive correlation only persisted for the women. Jones et al. [66] showed that total and upper-body LBM increased in junior and tier 3 senior female and male XCS athletes over 25 months, while an increase in lower-body LBM and a decrease in fat mass were observed in the women only. Moreover, valid projection models for distance performance (using best FIS points and changes in FIS points) were only observed for the women, with total body mass, LBM and fat mass being important variables for performance projection. A follow-up study by Jones et al. [67] revealed valid projection models for performance in XCS (FIS distance and sprint points) and BIA (IBU points) for female athletes only, with LBM important for the projection of female BIA performance. These findings suggest that body composition and long-term improvements in body composition (i.e., increased LBM and decreased fat mass) are important for performance, particularly among female XCS and BIA athletes.

Hegge et al. [68, 69] reported that tier 3–4 female XCS athletes had a lower proportion of their LBM located in their upper versus lower body compared to their male counterparts. This difference was suggested as the primary explanation for increased sex differences in power production and peak aerobic capacity during ski ergometer DP when using the whole-body, the upper-body only (i.e., seated DP with isolated upper-body work) and DP with the arms only (i.e., no trunk movement) [69]. Sex differences in upper-body power production were also augmented with increased exercise intensity (i.e., 88% during low-intensity submaximal poling, 95% during 3 min of maximal poling, 108% during 30 s of maximal poling and 118% during a peak power pull-down test) [68].

No relationships were observed between the longitudinal development of anthropometric variables and success in either female or male SpSk athletes [62]. While relatively little data are available for SpSk, no data on sex differences in anthropometric variables have been reported at all for NC or SkiMo athletes.

Overall, the observed sex differences in body composition align with findings from other endurance sports [6]. A recent review [70] examining the relationship between body composition and performance across various sports reported that increases in LBM positively influenced specific skills in racquet sports and endurance performance in XCS, NC, running and cycling. Additionally, the review found that body fat indices were negatively associated with performance in prolonged endurance events and average speed in some team sports. Specifically, findings from XCS and BIA suggest that increasing LBM, particularly in the upper body, and reducing fat mass can enhance performance, especially in female athletes [66, 67]. However, suggesting such changes must be approached individually, with high caution and in association with clinical expertise due to the potential physical and mental health risks associated with relative energy deficiency in sport (REDs) [71]. Future studies should investigate both the performance and health effects of long-term changes in body composition in female winter endurance athletes.

### Peak Oxygen Uptake

VO_2max_ and VO_2peak_ have been measured in OWG endurance sport athletes using a range of laboratory-based exercise modalities including cycle ergometry, treadmill running and roller skiing, and DP ski ergometry. Due to the utilization of diverse exercise modalities and test protocols, we have employed the term VO_2peak_ to reflect peak/maximal oxygen uptake throughout the following discussion. Measurements are typically reported in absolute terms (L·min^−1^) and/or relative to body mass (mL·kg^−1^·min^−1^) and, due to lower average body masses in women compared to men, sex differences are considerably smaller for relative versus absolute measurements of VO_2peak_.

**Fig. 2:**
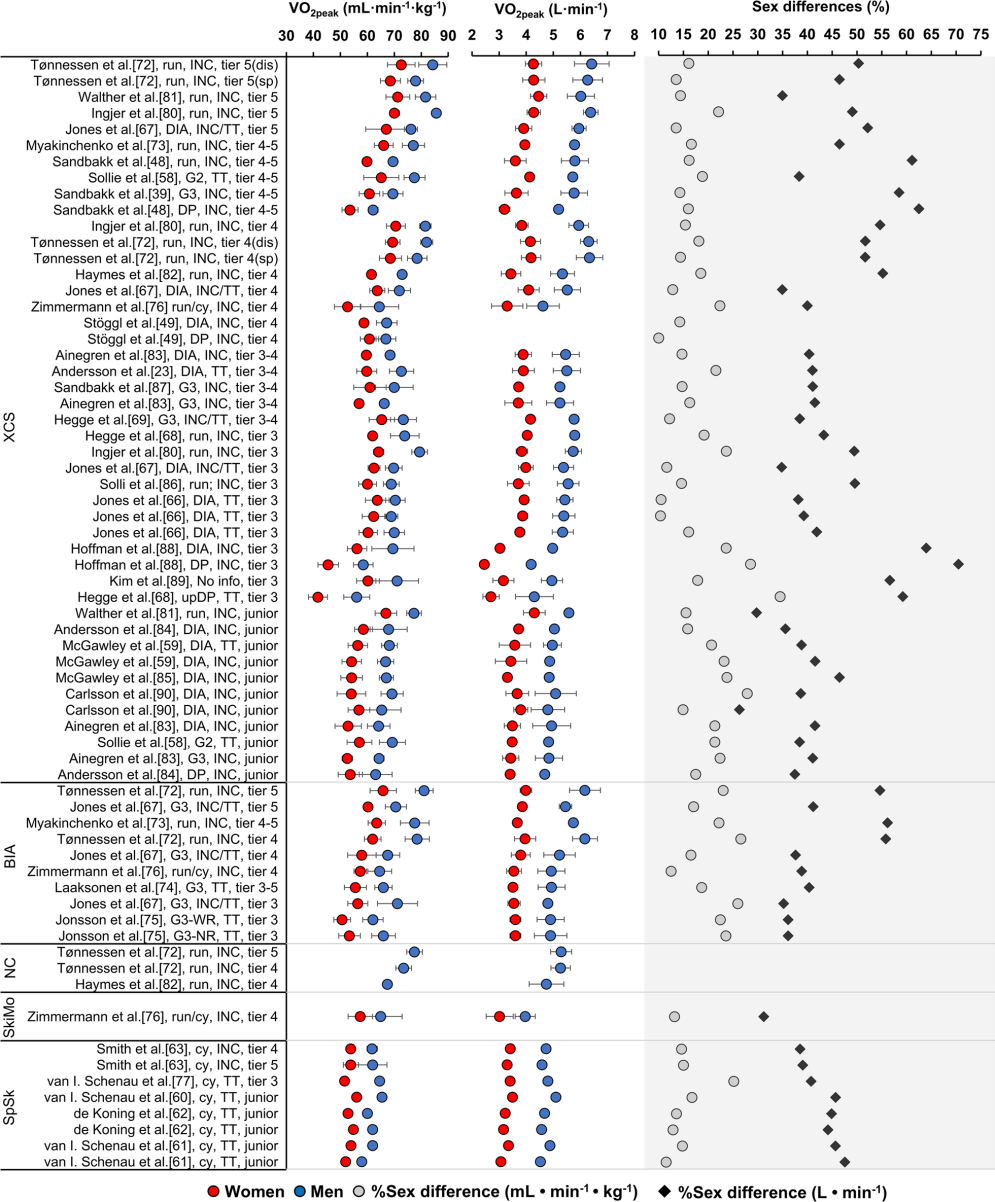
Overview of sex differences in relative and absolute peak oxygen uptake (VO_2peak_) values derived from 24 studies of cross-country skiing (XCS) [23, 39, 48, 49, 58, 59, 66–69, 72, 73, 76, 80–90], six studies of biathlon (BIA) [67, 72–76], two studies of Nordic combined (NC) [72, 82], one study of ski mountaineering (SkiMo) [76] and five studies of long-track speed skating (SpSk) [60–63, 77]. Studies are organized by sport, athlete tier level, athlete specialization (dis: distance; sp: sprint), exercise mode (run: running; cy: cycling; DIA: diagonal stride; G2: gear 2; G3: gear 3; DP: double poling; upDP: upper-body double poling; NR: no rifle; WR: with rifle) and test protocol (INC: incremental protocol; TT: time trial)

When running or treadmill roller skiing using different sub-techniques (DIA, DP, G2 or G3), sex differences in VO_2peak_ in junior and tier 3–5 senior XCS athletes have been reported to be 30–63% and 10–27% for absolute and relative measures, respectively (Fig. 2). Compared to XCS, fewer studies have compared the VO_2peak_ values of women and men in BIA. However, existing data demonstrate a similar pattern across the two sports, with significantly larger absolute and relative VO_2peak_ values recorded in men versus women by magnitudes of ∼ 36–56% and ∼ 18–27%, respectively, during treadmill running [72, 73] and treadmill roller skiing using the G3 skate technique [74, 75]. Tønnessen et al. [72] also reported data for male NC athletes, who demonstrated significantly lower absolute VO_2peak_ values compared to male XCS and BIA athletes. However, we are not aware of any sex comparisons of VO_2peak_ for NC. In SkiMo, one recent study of tier 3–4 athletes showed sex differences of 31% and 13% in absolute and relative VO_2peak_, respectively [76]. Comparable data were also reported for XCS and BIA athletes in that study, revealing sex differences in absolute and relative VO_2peak_ of 39% and 13–22%, respectively. A series of early studies in SpSk showed similar sex differences in absolute (39–48%) and relative (12–25%) VO_2peak_ values obtained during cycle ergometry [60, 62, 63, 77, 78]. The large range in sex differences reported for absolute VO_2peak_ values is related to the large differences (∼ 10–40%) in body masses across studies. The smallest sex differences in body mass and relative VO_2peak_ were typically observed in junior XCS athletes, while the largest sex differences in absolute values were observed in senior XCS sprinters.

Due to the greater use of DP in modern XCS racing, VO_2peak_ measured during DP has received increased attention in recent years. Sandbakk et al. [48] reported that male XCS sprint athletes obtained a higher VO_2peak_ in DP relative to their running VO_2peak_ compared to their female counterparts (89% and 86% for men and women, respectively). Similar findings were reported by Hegge et al. [68], where male XCS athletes utilized a higher proportion of their treadmill running VO_2peak_ during a 3-min upper-body DP performance test (76 and 67% for men and women, respectively). Furthermore, Hegge et al. [69] reported that the sex differences in the proportion of running VO_2peak_ attained during 3-min performance tests using whole-body DP, upper-body DP and isolated arm DP increased sequentially, which may be related to the greater proportion of muscle mass located in the arms of men. In contrast, a more recent study by Hansen et al. [79] reported no sex difference in the proportion of running VO_2peak_ attained during upper-body DP (72–73%). These authors suggested that a greater focus on upper-body strength and endurance training among female XC skiers in recent years may have closed this gap.

Overall, absolute and relative VO_2peak_ values have been reported to be 30–63% and 10–27% greater, respectively, in male compared to female OWG endurance sport athletes. Absolute differences are typically attributed to the smaller body size of women compared to men, while relative differences are related to lower hemoglobin concentrations and a higher relative fat mass in women [6]. The larger gap between the VO_2peak_ values attained during DP compared to running previously observed in female versus male XCS athletes seems to have diminished, likely due to increased knowledge about sex differences and more targeted training among women in recent years.

### Fractional Utilization of Peak Oxygen Uptake

Despite the importance of submaximal thresholds in endurance performance [91, 92], relatively few studies have compared the physiological responses to submaximal exercise between female and male OWG endurance sport athletes using standardized test protocols [39, 74, 93]. The lack of standardization when testing may be due to traditions within different laboratories, research groups and nations, and/or availability of equipment (e.g., a roller-ski treadmill). Moreover, the specific characteristics of Nordic skiing make methodological standardization particularly challenging compared to other exercise modes (e.g., cycling and running). As stated previously (in "Sub-technique selection in Nordic skiing" section), sub-technique choices in classic and skate skiing are largely determined by the skiing speed and gradient [18, 20, 23]. These factors in turn affect the physiological responses to exercise. Since different athletes will ski at different speeds and/or over different gradients for a given relative submaximal exercise intensity, or fractional utilization of VO_2peak_, different sub-techniques may be employed. This is particularly evident in cohorts of female and male athletes of similar ages and/or sex-specific performance levels, making sex comparisons difficult [18].

When comparing the responses at 4 mmol·L^−1^ of blood lactate concentration ([BLa]) during treadmill roller skiing in the G3 technique, Sandbakk et al. [39] showed that the fractional utilization of VO_2peak_ was higher for female (88%) compared to male (81%) tier 4 XCS sprinters. In contrast, Laaksonen et al. [74] reported no sex difference in the VO_2_ at 4 mmol·L^−1^ [BLa] expressed relative to VO_2peak_ (82 vs. 81% for women and men, respectively) in tier 3 biathletes employing the G3 technique during treadmill roller skiing. In support of these latter findings, Myakinchenko et al. [93] also showed the fractional utilization of VO_2peak_ at the second ventilatory threshold to be similar during running exercise for female and male XCS (88–89%) and BIA (85–87%) athletes. These results suggest that fractional utilization is not consistently higher for women versus men. Considering the chronology of the referenced articles it is possible that sex differences have declined over the last decade, especially in exercise modalities with large contributions from the upper body. However, the variation in training backgrounds and performance levels of the participants and/or testing methods (i.e., protocols and equipment) may also affect the differences between studies.

No sex comparisons for measures of fractional utilization appear to exist in NC, SkiMo or SpSk. In summary, relatively few studies have compared the physiological responses to submaximal exercise between female and male OWG endurance sport athletes and markers of fractional utilization have not typically been standardized. As such, the true sex differences in these variables are currently difficult to isolate. Further research employing standardized protocols and matching the performance levels of participants is warranted.

### Anaerobic Capacity

Direct quantification of the anaerobic energy yield during whole-body exercise is complicated and only possible via sophisticated, invasive, and expensive technologies. As such, a variety of indirect assessment methods have evolved, including the maximal accumulated oxygen deficit (MAOD) and gross efficiency (GE) methods [94]. Using the MAOD method, McGawley and Holmberg [59] calculated the accumulated oxygen deficit (AOD) of junior XCS athletes during a 600-m maximal treadmill TT using the diagonal roller-skiing technique. While no significant sex differences in AOD were observed (40.9 ± 9.5 and 47.3 ± 7.4 mL/kg for the female and male athletes, respectively), the authors suggested that the magnitude of the mean difference (6.4 mL/kg) and the moderate effect size reflected a likely practical difference between the sexes. In a more recent study of adolescent, junior and senior XCS athletes, Sollie et al. [58] reported AOD during a similar 3-min maximal treadmill TT using the G2 sub-technique. Again, no significant sex differences were observed, but moderate to large effect sizes indicated a potentially higher AOD in the male versus female athletes in all three age groups. In BIA, Laaksonen et al. [74] used the GE method to calculate AOD and found similar results during a 900-m (for women)/1000-m (for men) maximal treadmill TT using the G3 technique, whereby the sex differences were not significant (49.2 ± 9.9 and 57.7 ± 15.5 mL/kg for the female and male athletes, respectively) but the effect size was moderate.

In summary, a tendency for a higher AOD in male XCS and BIA athletes is evident, and this is likely related to the greater muscle mass relative to fat mass in men compared to women, which facilitates greater anaerobic energy turnover. However, the three original studies presented in this sub-section have all highlighted large standard deviations around the means, despite the homogeneous participant groups, which reflects the potential for measurement error when estimating AOD. No studies of sex differences in AOD are available for NC, SkiMo or SpSk.

### Efficiency of Movement

In tier 3–5 XCS and BIA athletes, GE increases at steeper inclines and has been shown to differentiate performance levels within both sexes [58, 83, 87, 95, 96]. Other studies have not related GE to athletic performance [24, 35]. Female and male XCS and BIA athletes matched for performance level (using FIS and IBU points) are normally not significantly different across different exercise modes, at least when corrected for differences in work rate between the sexes [39, 58, 69, 74, 87]. One study showed a 27% greater delta efficiency for female compared to male skiers during treadmill roller skiing using the DP sub-technique [88]. However, besides fundamental issues with interpreting delta efficiency [97], this study was performed on only four women and four men and performance levels were not matched between the sexes. Similar efficiency between sexes in XCS and BIA is consistent with data previously reported for summer endurance sports [3]. Of note, no efficiency data comparing sexes have been reported for the other OWG endurance sports.

## Sex Differences in Training

### Training Characteristics

Many studies have retrospectively reported training characteristics in endurance athletes, yet very few have compared women and men or provided material that can be used for valid sex comparisons of performance-matched athletes in OWG endurance sports. In a study of tier 4–5 biathletes, higher annual training volumes (821 vs. 728 h) were reported in women compared to men [93]. The women were reported to complete more strength and speed training, but a lower proportion of high-intensity training (HIT) and specific training (i.e., roller skiing and on-snow skiing). Similar findings were reported in a follow-up study with a larger sample of tier 4–5 XCS and BIA athletes, with a higher number of annual training days and significantly more training volume (864 vs. 843 h/year) in women compared to men [73]. In this study no sex differences were observed in gym strength training volume, but the women performed more upper body endurance training and speed training, and the men performed more moderate-intensity training (MIT) and HIT. Investigating the training characteristics of 17 tier 5 XCS athletes during their most successful season, Walther et al. [81] reported no sex differences in training volume (891 vs. 841 h/year) or intensity distribution, but consistent with Myakinchenko et al. [93], women engaged in more strength training compared to their male counterparts (75 vs. 41 h/year). The higher volume of strength training observed in female athletes could reflect an effort to compensate for their lower muscle mass compared to men, particularly in the upper-body [48]. As mentioned previously, this may be a relatively new training strategy based on improved knowledge and more targeted training among women in recent years. Indeed, in an earlier study of tier 3 XCS athletes, Hegge et al. [68] reported lower volumes of upper-body strength (and endurance) training in women compared to men.

Training comparisons are complicated due to differences in definitions and execution of training in various intensity zones (e.g., low, moderate, and high), variation in training forms (e.g., endurance, strength, and speed) and differences in exercise modes (e.g., on-/off snow skiing and cross training). Two studies have demonstrated that female XCS athletes record higher relative heart rate values when executing low-intensity training (LIT) compared to their male counterparts [85, 86]. These authors speculated that training on undulating terrain, and/or training alongside men, may elicit higher relative metabolic intensities among women, due to their lower average maximal velocities and aerobic capacities. Sex differences in sub-technique selection are observed during LIT and HIT [86], as have been observed during competition (i.e., more DIA and less DP among women; see "Sub-technique selection in Nordic skiing" section). Since the different sub-techniques load the upper and lower body to a different extent, this will induce different muscular loading in female and male athletes skiing over the same terrain during training.

Overall, there is a lack of sex comparisons of training characteristics across the different OWG endurance sports. The available data in XCS and BIA indicate higher strength training volumes in modern tier 4–5 female compared to male athletes, while no data on sex differences in training characteristics are available for NC, SkiMo or SpSK. Therefore, more research is necessary to gain greater insight into how training might be improved for female and male OWG endurance athletes.

### Training Adaptations

Only two studies in XCS have included information about sex differences in adaptations to training interventions. Investigating the effects of supplementary upper-body strength training (i.e., five different exercises with two sets of 5–8 repetitions maximum followed by core stability and 30–90 s sets of XCS-specific strength exercises) versus ski-ergometer training (i.e., 3–5 sets of 3–8-min intervals at 90–95% of HR_max_ followed by 5–10 sets of 10–60 s intervals at 95–100% of HR_max_) in junior female and male athletes, Carlsson et al. [90] reported that the female skiers in both groups improved their maximal treadmill DP roller skiing speed and VO_2max_, whereas only the male skiers in the strength group improved their maximal speed. Similar findings were reported by Kim et al. [89], who investigated the effects of 12 weeks of polarized training on body composition, cardiorespiratory function and upper-body power in tier 3 XCS and BIA athletes. Here, female athletes improved their 500-m ski ergometer DP performance to a greater degree than the men. However, it is uncertain whether the findings in these training studies reflect real sex differences in training adaptations, or differences in initial training or performance levels between the female and male participants. In summary, there is a lack of evidence regarding sex differences in adaptations to training within the OWG endurance sports, which is consistent with research in summer endurance sports.

## Sex Differences in Long-Term Athlete Development

Only five studies have provided data on potential sex differences in the long-term development of athletes within the OWG endurance sports and again, there is no information for NC or SkiMo. Investigating the relative performance progression (i.e., change in FIS points) in distance and sprint XCS athletes, Walther et al. [98] reported no sex differences in the age of peak performance in distance events (~ 26 years). However, while women showed the same peak age in sprint and distance events, men peaked ~ 0.8 years earlier than women in the sprint event. It was speculated that this was due to a higher degree of specialization towards sprint or distance events in men compared to women. Furthermore, men showed larger relative performance progression over the five years preceding peak age in both distance and sprint events. The authors speculated that this was due to a larger pool of male competitors at the top-level driving development.

To further understand athletic development from junior age to peak performance, Walther et al. [81] investigated changes in training characteristics and aerobic power from the most successful junior season to peak performance at senior level in tier 5 XCS athletes. They reported an increase of ~ 200 annual training hours over an average span of 8.5 years, with no sex differences between female and male athletes. Coinciding with this, the men increased their absolute and relative VO_2max_ (by ~ 8 and 6%, respectively), while women only increased their relative VO_2max_ (by ~ 7%). That women increase their relative VO_2max_ over time can be explained by reductions in total body and fat mass post adolescence [81]. This seems to be beneficial for performance among female XCS athletes, as changes in total body mass, fat mass and lean mass (as well as VO_2peak_ and speed at 4 mmol·L^−1^) have all been identified as important variables for projecting performance development in female (but not male) athletes [66]. Since female XCS athletes spend a larger proportion of their competition time in uphill terrain [18], and as relative VO_2max_ is more important when skiing uphill compared to flat sections, women may aim to maximize their relative VO_2max_ to a larger degree than men. Given the potential for a greater importance of reduced total body and fat mass, and increased lean mass among female athletes, it is paramount that healthy changes in body composition are prioritized throughout the long-term athlete development process (as suggested previously, in "Anthropometry" section).

In SpSk de Koning et al. [62] investigated developments in performance-determining variables from junior (16–17 years old) to senior (20–21 years old) level in high-performing athletes who either became “successful” (i.e., those who represented their country at the OWG, World or European Championships) or “unsuccessful” (i.e., those who did not represent their country at the highest level). No differences between the successful and unsuccessful athletes were evident at junior age, and at senior age there were no differences in anthropometric measures. The successful female SpSk athletes could produce a higher absolute power output (in W) during a 30-s cycle ergometer test compared to the unsuccessful women, while the successful male athletes could produce a higher absolute power output during a 2.5-min cycle ergometer test compared to their unsuccessful counterparts. The authors were unable to predict performance at senior age from measurements taken at junior age in either female or male athletes. This is supported by more recent evidence from Olympic sports [99] and has implications for talent identification and athlete development strategies.

Studies including information about sex differences in the long-term development of OWG endurance sport athletes are rare, with very few studies including direct comparisons of female and male athletes and no available data in NC and SkiMo. This is consistent with the sports science literature in general, where there is a lack of longitudinal studies characterizing the development of training, performance and performance-determining factors in endurance sport athletes [100].

## Methodological Considerations

Comparing performance and performance-determining factors between women and men in winter endurance sports is challenging, partly because the two sexes perform over different distances and/or durations, on different courses and over different terrains, and partly because it is difficult to match performance levels and training backgrounds. Aside from these factors, competitions are held in cold and unstable environments where snow properties are variable, which can complicate the evaluation of sex differences when women and men perform at different times and on different days. Other variables such as equipment, technical skills, pacing, metabolism, and thermoregulation are also difficult to compare in uncontrolled environmental conditions. Furthermore, the extent to which social, cultural, and economic factors continue to affect sex differences in sports performance is difficult to quantify [4, 34].

## Conclusions

This narrative review provides a detailed account of sex differences in performance and performance-determining factors in the OWG endurance sports. We found sex differences in competition speeds to be ~ 7–16% in XCS, ~ 12–16% in BIA and ~ 7–11% in SpSk. In XCS and BIA these differences were associated with more use of the DIA and G2 sub-techniques in women compared to men. Laboratory testing revealed 30–63% and 10–27% greater absolute and relative peak aerobic capacities, respectively, in male compared to female athletes. There is limited research pertaining to sex differences in training characteristics, although modern female XCS and BIA athletes tend to complete more strength training compared to their male counterparts. Overall, most available data on sex differences in OWG endurance sports are derived from studies in XCS, while there is a particular lack of studies investigating sex differences in NC and SkiMo. In conclusion, this review provides a comprehensive overview of sex differences in performance and performance-determining factors within and across the Olympic winter endurance sports and provides a scientific basis for designing training programs and future studies.

## Supplementary Information

Additional file1.

## Data Availability

All original data are freely available in the electronic supplementary material 1.

## Abbreviations

AOD: Accumulated oxygen deficit
BIA: Biathlon
[BLa]: Blood lactate concentration
CL: Cycle length
CR: Cycle rate
DIA: Diagonal stride
DK: Double poling with kick
DP: Double poling
FIS: International Skiing Federation
G2-5: Sub-technique gears 2 to 5
GE: Gross efficiency
HIT: High-intensity training
HR: Heart rate
HR_max_: Maximal heart rate
IBU: International Biathlon Union
INC: Incremental test protocol
ISMF: International Ski Mountaineering Federation
ISU: International Skating Union
LBM: Lean body mass
LIT: Low-intensity training
MAOD: Maximal accumulated oxygen deficit
MIT: Moderate-intensity training
NC: Nordic combined
OWG: Olympic Winter Games
REDs: Relative energy deficiency in sports
SkiMo: Ski mountaineering
SpSk: Long-track speed skating
TT: Time trial
VO_2max_: Maximal oxygen uptake
VO_2peak_: Peak oxygen uptake
XCS: Cross-country skiing
