# Correction: Sex Differences in Performance and Performance-Determining Factors in the Olympic Winter Endurance Sports

**DOI:** 10.1186/s40798-024-00805-6

**Published:** 2024-12-16

**Authors:** Guro Strøm Solli, Øyvind Sandbakk, Kerry McGawley

**Affiliations:** 1 Department of Sports Science and Physical Education, Nord University, Bodø, Norway; 2 School of Sport Science, UiT The Arctic University of Norway, Tromsø, Norway; 3 Department of Health Sciences, Swedish Winter Sports Research Centre, Mid Sweden University, Östersund, Sweden

**Correction: Sports Medicine - Open (2024) 10:126**

10.1186/s40798-024-00792-8

The original article contains an error in Table 1.

Under the description of SkiMo Mixed Relay, the cell displaying the text ‘Mass start, two women and two men’ should instead read: ‘One woman and one man, alternating, each skiing two laps’.

