# Supplementary material for: Sex Differences in Performance and Performance-Determining Factors in the Olympic Winter Endurance Sports
Source: Sports Med Open. 2024 Nov 20;10:126. doi: 10.1186/s40798-024-00792-8 (PMC11579258; doi:10.1186/s40798-024-00792-8)
Supplement: Supplementary file 1 — Additional file1. [file 40798_2024_792_MOESM1_ESM.pdf]

Electronic Supplementary Material

Sex Differences in Performance and Performance-Determining Factors in the Olympic Winter Endurance Sports

Sports Medicine

Guro Strøm Solli[1*], Øyvind Sandbakk[2], Kerry McGawley[3]

[1]Department of Sports Science and Physical Education, Nord University, Bodø, Norway

[2]Norwegian University of Science and Technology, Centre for Elite Sports Research, Department of Neuromedicine and Movement Science, Trondheim, Norway

[3]Swedish Winter Sports Research Centre, Department of Health Sciences, Mid Sweden University, Östersund, Sweden

*** Correspondence:** Guro Strøm Solli guro.s.solli@nord.no

Supplementary Table 1 Screened Papers

Authors	Year	XCS	BIA	NC	SkiMo	SpSk	Title
Björklund, G., Dzhilkibaeva, N., Gallagher, C., and Laaksonen, M.S.	2022		1				The balancing act between skiing and shooting – the determinants of success in biathlon pursuit and mass start events
Björklund, G., and Laaksonen, M.S.	2022		1				The Determinants of Performance in Biathlon World Cup Sprint and Individual Competitions
Björklund, G.	2018		1				Shooting efficiency for winners of World Cup and World Championship races in men's and women's biathlon: where is the cut-off?
Jonsson Kårström, M., Mcgawley, K., and Laaksonen, M.S.	2019		1				Physiological Responses to Rifle Carriage During Roller-Skiing in Elite Biathletes.
Jonsson Kårström, M., Stöggl, T., Lund Ohlsson, M., Mcgawley, K., and Laaksonen, M.S.	2022		1				Kinematical effects of rifle carriage on roller skiing in well-trained female and male biathletes
Jonsson Kårström, M., Staunton, C., Mcgawley, K., Björklund, G., and Laaksonen, M.S.	2023		1				Rifle carriage affects gear distribution during on-snow skiing in female and male biathletes.
Luchsinger, H., Kocbach, J., Ettema, G., and Sandbakk, Ø	2018		1				Comparison of the Effects of Performance Level and Sex on Sprint Performance in the Biathlon World Cup
Luchsinger, H., Kocbach, J., Ettema, G., and Sandbakk, Ø.	2019		1				The Contribution From Cross-Country Skiing and Shooting Variables on Performance-Level and Sex Differences in Biathlon World Cup Individual Races.
Laaksonen, M.S., Andersson, E., Jonsson Kårström, M., Lindblom, H., and Mcgawley, K.	2020		1				Laboratory-Based Factors Predicting Skiing Performance in Female and Male Biathletes
Sollie, O., and Losnegard, T.	2022		1				Performance and Micro-Pacing Strategies in a Freestyle Cross-Country Skiing Distance Race
Fornasiero, A., Callovini, A., Fornoni, S., Savoldelli, A., Schena, F., Holmberg, H.C., and Bortolan, L.	2023				1		Participation and performance by women and men in ski-mountaineering sprint races during the past decade
Fornasiero, A., Fornoni, S., Callovini, A., Todesco, B., Savoldelli, A., Schena, F., Holmberg, H.-C., Pellegrini, B., and Bortolan, L.	2023				1		Analysis of Sprint Ski Mountaineering Performance
De Boer, R.W.D., and Nilsen, K.L.	1989					1	Work per stroke and stroke frequency regulation in Olympic speed skating
De Boer, R.W., and Nilsen, K.L.	1989					1	The gliding and push-off technique of male and female Olympic speed skaters
De Koning, J.J., Bakker, F.C., De Groot, G., and Van Ingen Schenau, G.J.	1994					1	Longitudinal development of young talented speed skaters: physiological and anthropometric aspects.
Hofman, N., Orie, J., Hoozemans, M.J.M., Foster, C., and De Koning, J.J.	2017					1	Wingate test is a strong predictor of 1500 m performance in elite speed skaters
Muehlbauer, T., Panzer, S., and Schindler, C.	2010					1	Pacing pattern and speed skating performance in competitive long-distance events.
Muehlbauer, T., Schindler, C., and Panzer, S.	2010					1	Pacing and performance in competitive middle-distance speed skating
Muehlbauer, T., Schindler, C., and Panzer, S.	2010					1	Pacing and Sprint Performance in Speed Skating During a Competitive Season
Noordhof, D.A., Foster, C., Hoozemans, M.J.M., and De Koning, J.J.	2014					1	The association between changes in speed skating technique and changes in skating velocity
Peng, Q., Li, F., Liu, H., and Gomez, M.A.	2022					1	Analysis of Pacing Behaviors on Mass Start Speed Skating

Smith, D.J., and Roberts, D.	1991					1	Aerobic, Anaerobic and Isokinetic Measures of Elite Canadian Male and Female Speed Skaters
Stoter, I.K., Hettinga, F.J., Otten, E., Visscher, C., and Elferink-Gemser, M.T.	2020					1	Changes in technique throughout a 1500-m speed skating time-trial in junior elite athletes: Differences between sexes, performance levels and competitive seasons.
Van Ingen Schenau, G.J., and De Groot, G.	1983					1	Differences in oxygen consumption and external power between male and female speed skaters during supramaximal cycling.
Van Ingen Schenau, G.J., De Koning, J.J., Bakker, F.C., and De Groot, G.	1996					1	Performance-influencing factors in homogeneous groups of athletes: a cross-sectional study
Van Ingen Schenau, G.J., and De Groot, G	1983					1	On the origin of differences in performance level between elite male and female speed skaters
Van Ingen Schenau, G.J., Bakker, F.C., De Groot, G., and De Koning, J.J.	1992					1	Supramaximal cycling tests do not detect seasonal progression in performance in groups of elite speed skaters
Van Ingen Schenau, G.J., De Boer, R.W., Geysel, J.S., and De Groot, G.	1988					1	Supramaximal test results of male and female speed skaters with particular reference to methodological problems.
Ainegren, M., Carlsson, P., Tinnsten, M., and Laaksonen, M.S.	2013	1					Skiing economy and efficiency in recreational and elite cross-country skiers.
Andersson, E.P., Hämberg, I., Do Nascimento Salvador, P.C., and Mcgawley, K. (2021).	2021	1					Physiological responses and cycle characteristics during double-poling versus diagonal-stride roller-skiing in junior cross-country skiers.
Andersson, E.P., Govus, A., Shannon, O.M., and Mcgawley, K.	2019	1					Sex Differences in Performance and Pacing Strategies During Sprint Skiing.
Ardigò, L.P., Stöggl, T.L., Thomassen, T.O., Winther, A.K., Sagelv, E.H., Pedersen, S., Hammer, T.M., Heitmann, K.A., Olsen, O.-E., and Welde, B.	2020	1					Ski Skating Race Technique—Effect of Long Distance Cross-Country Ski Racing on Choice of SkatingTechnique in Moderate Uphill Terrain
Bolger, C.M., Kocbach, J., Hegge, A.M., and Sandbakk, Ø.	2015	1					Speed and heart-rate profiles in skating and classical cross-country skiing competitions.
Carlsson, T., Wedholm, L., Nilsson, J., and Carlsson, M.	2017	1					The effects of strength training versus ski-ergometer training on double-poling capacity of elite junior cross-country skiers.
Carlsson, M., Carlsson, T., Hammarström, D., Malm, C., and Tonkonogi, M.	2014	1					Prediction of race performance of elite cross-country skiers by lean mass.
Hansen, L.M., Sandbakk, Ø., Ettema, G.J.C., and Baumgart, J.K.	2021	1					Upper- vs. Lower-Body Exercise Performance in Female and Male Cross-Country Skiers.
Hegge, A.M., Myhre, K., Welde, B., Holmberg, H.C., and Sandbakk, Ø.	2015	1					Are Gender Differences in Upper-Body Power<br>Generated by Elite Cross-Country Skiers<br>Augmented by Increasing the Intensity of<br>Exercise?
Hegge, A.M., Bucher, E., Ettema, G., Faude, O., Holmberg, H.C., and Sandbakk, Ø.	2016	1					Gender differences in power production, energetic capacity<br>and efficiency of elite cross-country skiers during whole-body,<br>upper-body, and arm poling
Hoffman, M.D., Clifford, P.S., Watts, P.B., O'hagan, K.P., and Mittelstadt, S.W.	1995	1					Delta efficiency of uphill roller skiing with the double pole and diagonal stride techniques.
Jones, T.W., Lindblom, H.P., Karlsson, Ø., Andersson, E.P., and Mcgawley, K.	2021	1					Anthropometric, Physiological, and Performance Developments in Cross-country Skiers
Jonsson, M., Welde, B., and Stöggl, T.L.	2019	1					Biomechanical differences in double poling between sexes and level of performance during a classical cross-country skiing competition.
Kim, T.H., Han, J.K., Lee, J.Y., and Choi, Y.C.	2021	1					The Effect of Polarized Training on the Athletic Performance of Male and Female Cross-Country Skiers during the General Preparation Period.

Losnegard, T., Kjeldsen, K., and Skattebo, Ø.	2016	1					An analysis of the pacing strategies adopted by elite cross-country skiers.
Mcgawley, K., and Holmberg, H.-C.	2014	1					Aerobic and anaerobic contributions to energy production among junior male and female cross-country skiers during diagonal skiing.
Mcgawley, K., Juudas, E., Kazior, Z., Ström, K., Blomstrand, E., Hansson, O., and Holmberg, H.-C.	2017	1					No Additional Benefits of Block- Over Evenly-Distributed High-Intensity Interval Training within a Polarized Microcycle
Sandbakk, Ø., Hegge, A., and Ettema, G	2013	1					The role of incline, performance level, and gender on the gross mechanical efficiency of roller ski skating.
Sandbakk, Ø., Ettema, G., and Holmberg, H.C.	2012	1					Gender differences in endurance performance by elite cross-country skiers are influenced by the contribution from poling.
Sandbakk, Ø., Ettema, G., Leirdal, S., and Holmberg, H.C.	2012	1					Gender differences in the physiological responses and kinematic behaviour of elite sprint cross-country skiers.
Solli, G.S., Kocbach, J., Sandbakk, B.S., Haugnes, P., Losnegard, T., and Sandbakk, Ø	2020	1					Sex-based differences in sub-technique selection during an international classical cross-country skiing competition.
Solli, G.S., Kocbach, J., Seeberg, T.M., Tjonnas, J., Rindal, O.M.H., Haugnes, P., Torvik, P.O., and Sandbakk, Ø.	2018	1					Sex-based differences in speed, sub-technique selection, and kinematic patterns during low- and high-intensity training for classical cross-country skiing.
Sollie, O., and Losnegard, T.	2022	1					Sex Differences in Physiological Determinants of Performance in Elite Adolescent, Junior, and Senior Cross-Country Skiers.
Stöggl, T., Welde, B., Supej, M., Zoppirolli, C., Rolland, C.G., Holmberg, H.C., and Pellegrini, B.	2018	1					Impact of Incline, Sex and Level of Performance on Kinematics During a Distance Race in Classical Cross-Country Skiing.
töggl, T., Ohtonen, O., Takeda, M., Miyamoto, N., Snyder, C., Lemmettylä, T., Linnamo, V., and Lindinger, S.J	2019	1					Comparison of Exclusive Double Poling to Classic Techniques of Cross-country Skiing
Walther J, Haugen T, Solli GS, Tønnessen E, Sandbakk Ø.	2023	1					From Juniors to Seniors: Changes in Training Characteristics and Aerobic Power in 17 World-Class Cross-Country Skiers
Walther, J., Mulder, R., Noordhof, D.A., Haugen, T.A., and Sandbakk, Ø.	2021	1					Peak Age and Relative Performance Progression in International Cross-Country Skiers.
Jones, T.W., Lindblom, H.P., Laaksonen, M.S., and Mcgawley, K.	2023	1	1				Using multivariate data analysis to project performance in biathletes and cross-country skiers
Myakinchenko, E.B., Heil, D.P., Kriuchkov, A.S., Feofilaktov, V.V., Kuzmichev, V.A., and Adodin, N.V.	2022	1	1				Physiological profiles and training loads of international level male and female cross-country skiers and biathletes
Myakinchenko, E.B., Kriuchkov, A.S., Adodin, N.V., and Feofilaktov, V.	2020	1	1				The Annual Periodization of Training Volumes of International-Level Cross-Country Skiers and Biathletes
Tønnessen, E., Haugen, T.A., Hem, E., Leirstein, S., and Seiler, S.	2015	1	1	1			Maximal aerobic capacity in the winter-Olympics endurance disciplines: Olympic-medal benchmarks for the time period 1990-2013.
Zimmermann, P., Wüstenfeld, J., Zimmermann, L., Schöffl, V., and Schöffl, I.	2022	1	1		1		Physiological Aspects of World Elite Competitive German Winter Sport Athletes.
Haymes, E.M., and Dickinson, A.L.	1980	1		1			Characteristics of elite male and female ski racers.
Ingjer, F.	1991	1					Maximal oxygen uptake as a predictor of performance ability in women and men elite cross-country skiers.
